# Within-Neighborhood Patterns and Sources of Particle Pollution: Mobile Monitoring and Geographic Information System Analysis in Four Communities in Accra, Ghana

**DOI:** 10.1289/ehp.0901365

**Published:** 2010-01-07

**Authors:** Kathie L. Dionisio, Michael S. Rooney, Raphael E. Arku, Ari B. Friedman, Allison F. Hughes, Jose Vallarino, Samuel Agyei-Mensah, John D. Spengler, Majid Ezzati

**Affiliations:** 1 Harvard School of Public Health, Boston, Massachusetts, USA; 2 Harvard Initiative for Global Health, Cambridge, Massachusetts, USA; 3 Cyprus International Institute for the Environment and Public Health, Nicosia, Cyprus; 4 Department of Geography and Resource Development; 5 Department of Physics and; 6 Environmental Science Program, University of Ghana, Legon, Accra, Ghana

**Keywords:** Africa, biomass, geographic information system, particulate matter, poverty, spatial, urbanization

## Abstract

**Background:**

Sources of air pollution in developing country cities include transportation and industrial pollution, biomass and coal fuel use, and resuspended dust from unpaved roads.

**Objectives:**

Our goal was to understand within-neighborhood spatial variability of particulate matter (PM) in communities of varying socioeconomic status (SES) in Accra, Ghana, and to quantify the effects of nearby sources on local PM concentration.

**Methods:**

We conducted 1 week of morning and afternoon mobile and stationary air pollution measurements in four study neighborhoods. PM with aerodynamic diameters ≤ 2.5 μm (PM_2.5_) and ≤ 10 μm (PM_10_) was measured continuously, with matched global positioning system coordinates; detailed data on local sources were collected at periodic stops. The effects of nearby sources on local PM were estimated using linear mixed-effects models.

**Results:**

In our measurement campaign, the geometric means of PM_2.5_ and PM_10_ along the mobile monitoring path were 21 and 49 μg/m^3^, respectively, in the neighborhood with highest SES and 39 and 96 μg/m^3^, respectively, in the neighborhood with lowest SES and highest population density. PM_2.5_ and PM_10_ were as high as 200 and 400 μg/m^3^, respectively, in some segments of the path. After adjusting for other factors, the factors that had the largest effects on local PM pollution were nearby wood and charcoal stoves, congested and heavy traffic, loose dirt road surface, and trash burning.

**Conclusions:**

Biomass fuels, transportation, and unpaved roads may be important determinants of local PM variation in Accra neighborhoods. If confirmed by additional or supporting data, the results demonstrate the need for effective and equitable interventions and policies that reduce the impacts of traffic and biomass pollution.

Urban air pollution is responsible for an estimated 800,000 annual deaths worldwide (Cohen et al. 2004; Ezzati et al. 2002). The burden of disease from air pollution exposure is borne disproportionately by the growing urban populations in the developing world, where pollution is substantially higher than in high-income countries (Cohen et al. 2004). Research in cities in the United States and Europe has demonstrated substantial spatial variation in air pollution between and within neighborhoods, in relation primarily to specific sources as well as to neighborhood socioeconomic status (SES) (Buzzelli and Jerrett 2004; Charron and Harrison 2005; Hoek et al. 2001, 2002; Holmes et al. 2005; Kinney and O’Neill 2006; Levy et al. 2000, 2001; Loh et al. 2002; O’Neill et al. 2003; Su et al. 2007, 2008; Weijers et al. 2004).

Sources of air pollution in developing country cities include those that are common in high-income nations (e.g., transportation and industrial pollution), as well as biomass and coal fuel use for household and commercial purposes (Barnes et al. 2005) and resuspended dust from unpaved roads. A few studies have examined spatial variability and sources of air pollution in cities in developing countries (Chowdhury 2004; Engelbrecht et al. 2001; Etyemezian et al. 2005; Jackson 2005; Padhi and Padhy 2008; Saksena et al. 2003; van Vliet and Kinney 2007; Zheng et al. 2005), but few have been in low-income “slum” neighborhoods, have systematically examined variation in air pollution within neighborhoods, or have analyzed the effects of sources on local pollution levels. The absence of data on air pollution in urban communities in the developing world, especially from slums, occurs despite the evidence that other environmental factors, such as sanitation infrastructure, are worse in slum areas (Sclar et al. 2005; Songsore and McGranahan 1998).

In the present study we systematically collected and analyzed data on particulate matter (PM) in four neighborhoods in Accra, Ghana, with emphasis on within-neighborhood variability of PM pollution in relation to nearby sources. Accra is a major city in sub-Saharan Africa, the region with the highest urban population growth rate in the world (United Nations Department of Economic and Social Affairs 2004).

## Materials and Methods

### Study location

Accra is the capital city of Ghana and is located on the Gulf of Guinea, with an elevation of 0–60 m above sea level. The population of the Accra metropolitan area (AMA) increased from 600,000 in 1970 to 1 million in 1984 and 1.7 million in 2000.

**Figure 1:**
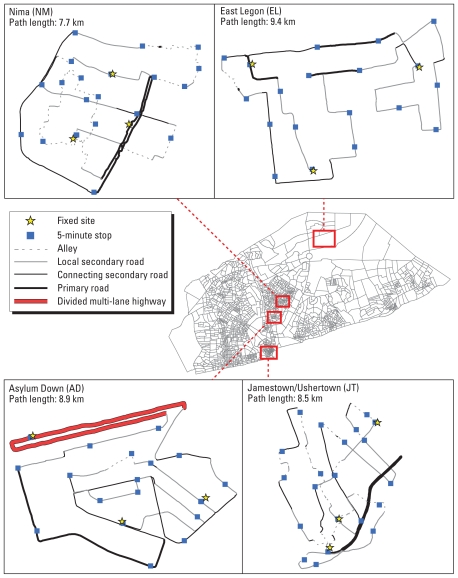
Map of Accra metropolitan area, study areas, and mobile monitoring path, delimited by census enumeration areas (EAs). EAs have nearly the same population; hence the area of an EA is inversely related to population density. Local secondary roads are smaller roads whose traffic is primarily for the purpose of reaching a local destination; connecting secondary roads are smaller roads used for passing through the neighborhood.

Our study took place in four Accra neighborhoods: Jamestown/Ushertown (JT), Asylum Down (AD), Nima (NM), and East Legon (EL) (Figure 1). Study neighborhoods were selected such that they lie on a nearly straight line from the coast to the northern boundaries of the AMA, and they had varying SES based on data from the 2000 Population and Housing Census of Ghana (Agyei-Mensah and Owusu 2009). JT is an old inner-core area that lies between the coast and the Accra business center; AD and NM are located approximately 3 km inland, separated from one another by the Ring Road Central; EL is 10 km inland and lies just north of Kotoka International Airport in Accra. JT and NM are poor, densely populated communities where many residents live in shared compounds along narrow alleys. Biomass is the predominant fuel used for household cooking and is also used for small-scale commercial purposes, such as the smoking of fish over wood fires (JT) and cooking of food by street vendors (JT and NM). Both JT and NM have markets and many small vendors that sustain activities throughout the day. A large, busy road with a central bus station runs through NM. AD is a middle-class neighborhood, with a combination of residential and commercial buildings. It is bordered by the Ring Road Central, one of the largest and busiest roads in Accra. Street food vendors are less common in AD than in JT and NM. EL is an upper-class, sparsely populated, residential neighborhood with most families living in modern homes on large plots of land. The streets are quiet during the day. The main road in EL has heavier traffic primarily during the morning and evening commute periods. According to data from the Ghana 2000 Population and Housing Census (unpublished data), 81% of households in JT, 75% in NM, 49% in AD, and 46% in EL use charcoal and/or wood as their primary cooking fuel.

### Study design

A study of spatial and temporal variability of air pollution would ideally be based on continuous data using a dense network of monitors placed at multiple locations, with additional information on sources and meteorologic factors gathered at each location. For PM, which is generally considered the best indicator of the health effects of urban air pollution, such an approach would be prohibitively costly and logistically difficult using current technologies, especially when continuous (vs. temporally integrated/averaged) data are needed. We used a combination of mobile and stationary monitors to examine the spatiotemporal patterns of air pollution and the effects of sources in these neighborhoods.

We conducted consecutive days of mobile monitoring in each neighborhood (7 days in most neighborhoods) (Table 1). There were two monitoring tours on each day, unless it rained for more than approximately 30 min or there was an equipment failure, in which case the tour was postponed or cancelled. In each monitoring tour, we walked slowly along a predetermined path recording data at 1-min intervals with a continuous real-time PM monitor and a global positioning system (GPS) unit. The path for each neighborhood was designed to traverse different areas, including main highways/roads, local roads, residential alleys and foot paths, and markets. The paths ranged 7.7–9.4 km in length, and the tours lasted 4.5–5.5 hr in duration in different neighborhoods (Figure 1). Each path included approximately 20 predetermined 5-min stops, including one stop at ground level in front of most fixed-monitoring sites (Figure 1). At each stop, information on PM sources within 10–15 m was recorded through a visual sighting of the source or presence of smoke using a standardized form programmed into Palm Z22 handheld units (Palm, Inc., Sunnyvale, CA, USA). The information included nearby biomass stoves (including fuel type and number of stoves), traffic flow, other combustion sources (e.g., trash burning), and road surface.

We also measured 48-hr integrated concentrations of PM with aerodynamic diameters ≤ 2.5 μm (PM_2.5_) and ≤ 10 μm (PM_10_) at three roof-top fixed-monitoring sites in each neighborhood [two in NM because an earlier pilot study found nearly identical concentrations at two of the original three sites (Arku et al. 2008)]. One fixed-monitoring site in each neighborhood was located along a main road, and the others were located in residential areas (> 100 m from main roads, although some were along smaller roads). The monitors were 4–7 m above ground level so that the air was relatively well mixed and less likely to be strongly affected by a source in the immediate vicinity. We also measured PM_2.5_ and PM_10_ continuously at as many fixed-monitoring sites as possible. Analysis of between-neighborhood variation using data from the fixed-monitoring sites are presented elsewhere (Dionisio et al. 2010).

Measurements were done in April 2007 (before the main rainy season) in AD and in July–August 2007 (after the main rainy season) in the other neighborhoods. There were no unusual meteorologic factors during the measurement periods.

### PM measurement methods

We used DustTrak model 8520 monitors (TSI Inc., Shoreview, MN, USA) for continuous measurement of PM_2.5_ and PM_10_. DustTrak has an internal laser photometer that uses a 90° light-scattering laser diode to measure PM concentration in air drawn by an internal pump. DustTrak monitors were operated at 1.7 L/min and used TSI-supplied inlet nozzles with a cutoff of 10 μm (aerodynamic diameter) for PM_10_ measurement; for PM_2.5_ measurement, DustTrak monitors were fitted with an external size-selective inlet containing a level greased impaction surface and with a cutoff of 2.5 μm (aerodynamic diameter) and were operated at 0.8 L/min. The DustTrak measures PM concentrations every second and was set to average these measurements and record at 1-min intervals. The DustTrak monitors were set to zero against a zero filter on each measurement day. The factory-specified resolution of the DustTrak monitor is ± 0.1% of reading or ± 1 μg/m^3^, whichever is greater.

PM measured using light-scattering technologies is subject to error because DustTrak photometers are calibrated to aerosols whose characteristics (e.g., shape, size, density, and refractive index) may differ from those in our study and because measured concentration may be affected by factors such as humidity. For the same reasons, measurement errors can vary across days or neighborhoods. To adjust for DustTrak measurement error, we corrected continuous (DustTrak) PM concentrations by a correction factor (CF) calculated using the gravimetric data as described below. PM concentration measured gravimetrically has substantially less measurement error but, by definition, measures the average concentration for the whole measurement period. Gravimetric PM measurements are described in detail elsewhere (Dionisio et al. 2010). In summary, gravimetric PM samples were collected on a PTFE (polytetrafluoroethylene) filter with ring (Teflo, 0.2 μm pore size, 37 mm diameter; Pall Life Sciences, Port Washington, NY, USA), back-supported by a Whatman drain disc (Whatman Inc., Piscataway, NJ). PM_10_ measurements used a Harvard impactor (Marple and Willeke 1976; Marple et al. 1987) with a D_50_ (50% collection efficiency) of 10 μm (aerodynamic diameter) at 4 L/min (± 10%), with two consecutive pre-oiled impactor plates serving as the impaction surface. PM_2.5_ measurements used a modified Harvard impactor combined with a polyurethane foam (PUF) PM_2.5_ size-selective inlet, with a D_50_ of 2.5 μm at 5 L/min (±10%), with a PUF pad serving as the impaction surface. All PM concentrations were blank corrected.

The CF was calculated separately for PM_2.5_ and PM_10_ and for each 48-hr measurement period. At each fixed-monitoring site we used the gravimetric-to-DustTrak ratio as the CF for the site itself. The CF for DustTrak monitors used in mobile monitoring was calculated as the geometric mean of the CFs of the corresponding size fraction for all the fixed-monitoring sites in that neighborhood on the measurement day. The mean and 5th and 95th percentiles of the individual fixed-site CFs were 0.75, 0.35, and 1.19, respectively; those of the mobile monitor CFs were 0.78, 0.48, and 1.25, respectively. Because of interruptions of either the gravimetric or the photometer measurements in JT, we had only a single CF for this neighborhood. We conducted the following sensitivity analyses to ensure that the missing CFs did not affect our overall conclusions: applying CFs calculated from the sites in NM, where measurements took place on the same days; and repeating all analyses with uncorrected JT fixed-site and mobile-monitor data, as has been done in at least one previous study without gravimetric data (Levy et al. 2001). The results from these two analyses were within the 95% confidence interval (CI) of each other, indicating that the conclusions are robust.

### Meteorologic/weather variables

PM concentration depends on meteorologic/weather variables such as humidity, wind speed, and recent rain. For this reason, we adjusted the estimated effects of nearby sources on local PM for these variables. Data on relative humidity (RH), wind speed, and time since last rain were from a station near the Kotoka International Airport and maintained by the U.S. Department of Commerce National Oceanic and Atmospheric Administration (http://www.ncdc.noaa.gov/oa/climate/stationlocator.html). We predicted RH and wind speed for hours with missing data using simple linear models when data were missing for < 3 hr. When > 3 hr of data were missing, we used the average of RH for the same hour during 5 days before and 5 days after the missing value. We then fit a cubic spline function to hourly RH and wind speed to obtain RH values for each minute. RH and wind speed on days that we conducted mobile monitoring were on average 79% (interquartile range, 72–86%) and 11 miles/hr (interquartile range, 8–14 miles/hr).

### Data management

Using the time stamps of DustTrak monitors, we compiled error-corrected continuous PM_2.5_ and PM_10_ data from fixed sites and mobile monitors into a single data set, with each record representing a unique date, minute, and location. Geographic coordinates for each mobile monitor data point were also included using the GPS date/time stamp. Because GPS units measure true location with error, GPS coordinates were “snapped” to the nearest point on the monitoring path; the location of the path was ascertained using a Trimble GeoXT GPS unit (Trimble Navigation Limited, Sunnyvale, CA, USA) with a nominal error of < 1 m. When a point was at or near the intersection of two roads that were both on the path, the snapping retained the temporal ordering of data points. The average change in the position of points snapped to the path was about 3 m. Weather variables were also added using date and time stamps. We merged DustTrak and Palm date and time stamps and GPS geographic coordinates to identify the measurements taken at each stop along the monitoring path. Measurements were averaged over the approximately 5 min of recordings for each stop visit and then merged with the PM source data collected on the Palm units.

### Statistical analysis

We provide graphical presentation as well as descriptive statistics for mobile PM measurements. We also analyzed the stop data, which included simultaneous data on PM and sources at the same location, for the effects of specific pollution sources and meteorologic factors. All analyses used PM data corrected for DustTrak measurement error as described above.

We used regression analysis to quantify the effects of sources on PM concentrations at stops. Because the mobile monitors reached stops at different times, the PM data from mobile monitors alone cannot determine if observed variations across stops are due to sources or due to changes in the overall neighborhood PM level, which happened to be lower or higher when the monitor reached a specific stop. For example, if woodstoves were disproportionately present at stops visited early in the day in a specific neighborhood, and if other factors (e.g., heavy traffic) led to high PM concentration at those times in that neighborhood, the effects of woodstoves on PM might be overestimated. To deal with this issue, we adjusted the local PM-source regressions for the average neighborhood PM concentration at the same time; average neighborhood concentration was estimated as the average of those at all fixed-monitoring sites (which, by being fixed, vary only in time). This approach may, however, lead to an underestimation of the effects of sources if the neighborhood average itself was influenced by that source and others like it. For example, if the high neighborhood PM concentrations in early mornings resulted from the use of woodstoves, which happened to be present at stops visited at those hours, adjusting for neighborhood average could underestimate the role of woodstoves. Therefore, we present our regression results both with and without adjustment for the neighborhood average. We estimated the following regression equation:


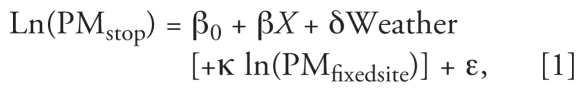


where *X* is a vector of source variables (data collected using Palm units); Weather is a vector of weather variables; β, δ, and κ are regression coefficients; and ɛ is an error term. We used a linear mixed effects model with a random group effect for each neighborhood-day (Davidian and Giltinan 1995; Laird and Ware 1982). Neighborhood-day group effect helps remove the influence of unobserved factors that affect all measurements in each neighborhood on the measurement day, for example, unmeasured weather pattern or phenomena that lead to more or less combustion. PM concentrations were log-transformed to ensure that model residuals were normally distributed.

The five measurements at each stop were averaged because source data were recorded once for the 5-min duration of the stop, and because averaging reduces random error due to short-term fluctuations in PM. We also smoothed the fixed-site continuous PM data to retain salient temporal patterns and remove minute-to-minute stochastic noise, which is likely to be highly local. We used a nonparametric regression [locally weighted scatterplot smoothing (LOWESS) regression] for smoothing, with a 60-min bounding radius, which tends to eliminate perturbations sustained for < 10 min but maintain patterns lasting more than 30 min (Cleveland et al. 1992).

All analyses were done separately for PM_2.5_ and PM_10_ using the open-source statistical analysis package R, version 2.6.1 (R Project for Statistical Computing, Vienna, Austria).

## Results

**Figure 2:**
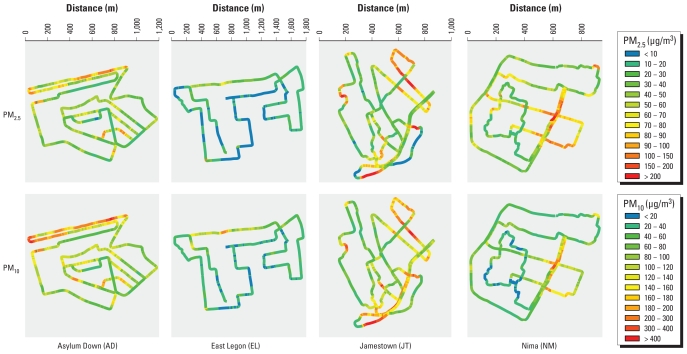
Concentrations of PM_2.5_ and PM_10_ along the walking paths in the study neighborhoods. For each neighborhood and PM size fraction, data from all monitoring days/tours were combined in a moving average, with a 50-m averaging interval.

Figure 2 shows the gravimetric-corrected concentrations of PM_2.5_ and PM_10_ along the walking path, averaged over all monitoring days/tours. In our measurement campaign, the EL walking path had the lowest levels of PM and the JT walking path the highest, with geometric means of PM_2.5_ and PM_10_ of 21 and 49 μg/m^3^, respectively, along the EL path and 39 and 96 μg/m^3^, respectively, along the JT path. In fact, the less polluted segments of the JT walking path had PM_2.5_ and PM_10_ values that were similar to the average for all of EL. AD and NM walking paths had PM pollution levels that fell between the other two neighborhoods, with geometric means of PM_2.5_ and PM_10_ of 35 and 86 μg/m^3^ for AD and 41 and 58 μg/m^3^ for NM. In AD and NM, pollution was highest along the largest roads/highways. Our observations during data collection indicate that the primary pollution source along the main highway in AD was traffic (cars, minibuses, and trucks) and in NM a combination of traffic and roadside biomass use.

**Figure 3:**
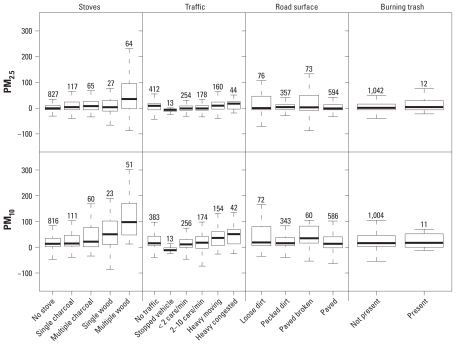
Crude associations of residual PM, defined as the difference between PM measured at stops and the neighborhood average, with sources present at the stop. In each plot, the middle line shows the median, and the bottom and top of the box show the 25th and 75th percentiles of data. Numbers indicate the number of stops contributing to each box plot.

Figure 3 shows the crude associations of nearby sources with residual PM, defined as the difference between PM measured during 5-min stops and the neighborhood average in the same 5 min. PM_2.5_ and PM_10_ measurements at stops with multiple woodstoves were, respectively, 30 μg/m^3^ and 85 μg/m^3^ higher than the neighborhood average at the same time (median residual); the residual PM_2.5_ and PM_10_ were smaller for stops that had one woodstove (8 μg/m^3^ and 32 μg/m^3^) or one or more charcoal stoves. When a stop had no stoves, residual PM_2.5_ and PM_10_ were only 0 μg/m^3^ and 14 μg/m^3^. Similarly, we generally found a gradient of residual PM pollution with increasing local traffic density. Residual PM_2.5_ and PM_10_ at stops near congested traffic were, respectively, 12 and 46 μg/m^3^ greater than the same metric for stops near light traffic (< 2 cars/min). However, residual PM_2.5_ (but not PM_10_) at stops with no traffic was higher than at stops with light and medium traffic (see below).

**Figure 4:**
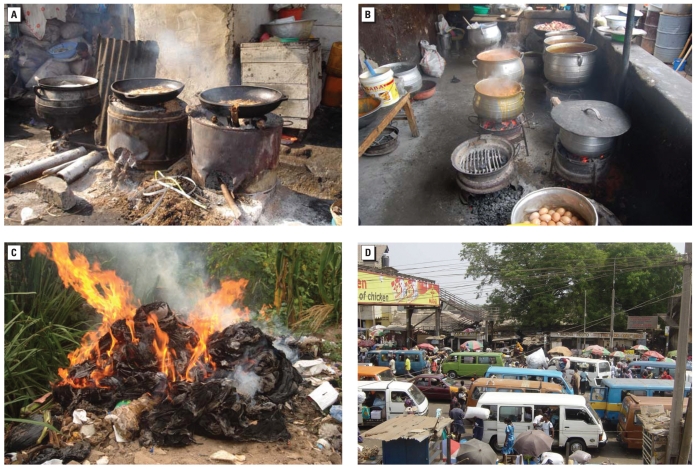
Woodstoves (*A*), charcoal stoves (*B*), trash burning (*C*), and congested traffic (*D*) in study neighborhoods.

The results in Figure 3 show only crude associations; that is, they do not consider the possibility that some sources may be more/less likely to co-occur at the same place. For example, it may be the case that stops with no traffic had higher PM_2.5_ because charcoal or wood stoves were present near them. Table 2 shows the adjusted association of PM with sources and demonstrates a number of features of local PM pollution in these neighborhoods. First, adjusting for average neighborhood pollution had some, but limited, influence on either the magnitude of the effects of individual sources or their statistical significance. Second, the presence of multiple woodstoves (Figure 4) had the unequivocal largest effect on nearby PM_2.5_ and PM_10_ concentrations. In the log-transformed model, multiple nearby woodstoves would be associated with nearly three times (297%; 95% CI, 247–357%) higher PM_2.5_ levels and more than two times (227%; 95% CI, 189–272%) higher PM_10_ levels after adjustment for all other source and meteorologic variables (262% and 197% higher if neighborhood averages were also adjusted for). The next most important determinants of local pollution were the presence of a single woodstove or multiple charcoal stoves, heavy/congested traffic, having loose dirt road surface, and trash burning (Figure 4). The coefficients of trash burning were significant only for PM_2.5_, possibly because this source was present at fewer stops than were other combustion sources. The coefficients of nearby stoves were ordered with larger effects from woodstoves than from charcoal stoves and larger effects from multiple stoves than from single ones. Further, for each stove category, the coefficients of the log-transformed regression were larger for PM_2.5_ than for PM_10_, that is, larger proportional effects on PM_2.5_. The coefficients of nearby traffic flow also rose monotonically with apparently comparable proportional effects on PM_2.5_ and PM_10_. Stops where road surface was loose dirt had significantly higher PM_2.5_ and PM_10_ concentrations, and those with broken paved surface had higher PM_10_ concentrations after adjustment for combustion sources; PM did not seem to vary with other road surface materials. The slightly larger proportional effects of loose dirt road surface on PM_2.5_ compared with PM_10_ is unexplained and may be attributable to the presence of unrecorded sources (e.g., stoves inside homes that were not visible to us). After controlling for traffic and other local sources at stops, the coefficient of distance from the main road was nonsignificant in most models.

## Discussion

This study provides one of the first systematic measurements showing how PM pollution varies within neighborhoods of varying SES in a developing country city and the role of specific combustion sources in local pollution patterns. Our results showed significant spatial variability in PM concentrations within a small geographic area in these neighborhoods (each ~ 1–2 km diameter). In our measurement campaign, the walking path in the lower SES neighborhood of JT had the highest pollution, followed by segments of the path along the primary road in NM and the Ring Road Central in AD. PM_2.5_ and PM_10_ were as high as 200 and 400 μg/m^3^, respectively, in some segments of the path.

Combinations of stationary and mobile measurements have been used to investigate variations in air pollution levels in relation to important sources in high-income countries (Larson et al. 2007; Levy et al. 2001; Su et al. 2007; Weijers et al. 2004). Our study is an innovative application of such a design by conducting measurements in a developing country city, choosing multiple neighborhoods with varied SES, and assessing the role of sources. Prior studies of local PM pollution have used different metrics (e.g., particle count vs. particle mass; fine vs. ultrafine particle mass). Therefore, our results can be compared only with selected other studies that measured PM_2.5_ and PM_10_ in urban microenvironments. This comparison shows that during this campaign PM pollution along primary roads was comparable to or higher than the most polluted urban microenvironments, for example, in buses and trolleys and near bus stations (Levy et al. 2000, 2001), and substantially higher than those in wood burning areas of the Pacific Northwest or roadside sites in European cities (Harrison et al. 2004; Hoek et al. 2002; Su et al. 2007). We could not locate other studies of small-area spatial variability and sources in developing country cities for direct comparison. More broadly, studies in Nairobi, Kenya, and Bolpur, India, found higher PM along major traffic routes than in nontraffic areas (Padhi and Padhy 2008; van Vliet and Kinney 2007), but these studies did not examine the presence of nontransportation combustion sources; a study at multiple sites in Addis Ababa, Ethiopia, also found spatial variation in short-term PM measurements but did not collect data on nearby sources (Etyemezian et al. 2005).

Our study has a number of innovations and strengths. We used a combination of georeferenced pollution and source data from mobile monitoring to investigate both the within-neighborhood spatial patterns of PM_2.5_ and PM_10_ pollution and the effects of nearby sources on local pollution. The data were from four neighborhoods that covered the full range of community SES in Accra. We used a combination of fixed-site and mobile-monitor continuous data to account for the background temporal pattern of air pollution that may confound the data from mobile monitors. Finally, we used integrated PM measurement to correct for the measurement error of continuous data measured with DustTrak monitors.

The data used in this study also have a number of limitations. First, data were collected during about 1 week in each neighborhood. The measurements in three neighborhoods (JT, NM, and EL) were conducted within a few weeks, and in the fourth (AD) a few months prior. Although there were no unusual meteorologic factors during data collection, it would be ideal to have multiple measurement campaigns in each neighborhood, in different seasons. Because of lack of data from different seasons, our results should not be used to estimate the usual or average pollution in these neighborhoods. However, our analysis of the effects of sources on local PM are unlikely to be affected by macro-level PM changes because we adjusted for average neighborhood pollution from fixed sites and used a mixed-effects model with neighborhood-day group effect. Second, PM measured with DustTrak monitors is subject to error. Although we systematically applied a CF to PM data, the steps involved in calculating CFs introduce additional uncertainty. Third, using mobile monitoring alone did not allow us to separate temporal and spatial changes in pollution. We relied on continuous PM data at fixed sites to adjust for temporal changes in neighborhood PM. If low-cost and low-power PM monitors were available, it would be ideal to have a large number of stationary monitors in the neighborhood instead of mobile ones.

## Conclusions

We found that, after adjusting for other factors, the factors wood and charcoal stoves, congested and heavy traffic, and trash burning had large and significant effects on local PM pollution in these Accra neighborhoods. Biomass fuels are a source of energy for households and small commercial purposes in urban sub-Saharan Africa, especially in low-income and marginalized neighborhoods (Bailis et al. 2005; Barnes et al. 2005); older vehicles are also common in sub-Saharan African cities. If other studies in Accra and other developing country cities show that the effects of these common sources on local pollution observed in our measurement campaign are typical of their usual contributions, there is need to identify and implement effective and equitable transportation regulations and policies that reduce the impacts of traffic pollution, and technological and policy innovations that can reduce air pollution from biomass fuels without restricting what may be the only energy source available to poor households.

## Figures and Tables

**Table 1:** Data used for the analysis, by PM size fraction and neighborhood.

	Neighborhood			
PM size fraction	AD	EL	JT	NM
PM_2.5_				
No. of mobile monitoring tours^a^	14	13	12	13
Total no. of stops contributing data to the regression analysis	279	247	288	286
No. of fixed sites contributing data to neighborhood average	3	3	1	2

PM_10_				
No. of mobile monitoring tours^a^	14	14	10	13
Total no. of stops contributing data to the regression analysis	279	265	240	286
No. of fixed sites contributing data to neighborhood average	2	1	1	1

a There were two tours on most days unless one was postponed or canceled due to rain or equipment malfunction.

**Table 2:** Regression coefficients (95% CIs) for multivariate analysis of the association of PM with sources, road surface, and meteorologic covariates.

	Model 1		Model 2	
Variable	Coefficient	*p*-Value	Coefficient	*p*-Value
Dependent variable: ln(PM_2.5_)				
Constant	−0.169 (−0.846 to 0.508)	0.63	0.897 (0.190 to 1.603)	0.013
Ln(neighborhood average)	0.522 (0.422 to 0.623)	< 0.001		
Distance to nearest main road (km)	−0.061 (−0.342 to 0.221)	0.67	0.044 (−0.244 to 0.332)	0.764
Trash burning	0.414 (0.082 to 0.746)	0.02	0.465 (0.118 to 0.812)	0.009
Traffic flow				
No traffic	0.0	NA	0.0	NA
Idling vehicle	−0.171 (−0.510 to 0.168)	0.32	−0.214 (−0.568 to 0.141)	0.24
Light (< 2 cars/min)	0.095 (−0.012 to 0.202)	0.08	0.097 (−0.014 to 0.209)	0.09
Medium (< 10 cars/min)	0.181 (0.059 to 0.302)	0.004	0.174 (0.047 to 0.300)	0.007
Heavy moving	0.326 (0.185 to 0.468)	< 0.001	0.339 (0.192 to 0.487)	< 0.001
Congested/stopped heavy traffic	0.466 (0.260 to 0.673)	< 0.001	0.496 (0.280 to 0.711)	< 0.001
Biomass stoves				
No stove	0.0	NA	0.0	NA
Single charcoal stove	0.153 (0.038 to 0.269)	0.009	0.155 (0.035 to 0.275)	0.01
Multiple charcoal stoves	0.294 (0.144 to 0.445)	< 0.001	0.313 (0.156 to 0.469)	< 0.001
Single wood stove	0.373 (0.134 to 0.613)	0.002	0.365 (0.116 to 0.614)	0.004
Multiple wood stoves	0.962 (0.783 to 1.142)	< 0.001	1.089 (0.905 to 1.273)	< 0.001
Road surface				
Paved	0.0	NA	0.0	NA
Paved broken	0.119 (−0.054 to 0.292)	0.18	0.161 (−0.019 to 0.342)	0.08
Packed dirt	0.009 (−0.095 to 0.114)	0.86	0.029 (−0.080 to 0.138)	0.60
Loose dirt	0.337 (0.175 to 0.498)	< 0.001	0.384 (0.216 to 0.552)	< 0.001
Meteorologic factors				
Wind speed (miles/hr)	−0.013 (−0.027 to 0.002)	0.09	−0.043 (−0.057 to −0.029)	< 0.001
RH	2.216 (1.600 to 2.832)	< 0.001	3.415 (2.806 to 4.024)	< 0.001
Ln(hours since rain + 1)	0.049 (−0.046 to 0.145)	0.31	0.089 (−0.023 to 0.201)	0.12

Dependent variable: ln(PM_10_)				
Constant	0.456 (−0.101 to 1.012)	0.11	1.582 (0.917 to 2.248)	< 0.001
ln(neighborhood average)	0.531 (0.455 to 0.606)	< 0.001		
Distance to nearest main road	−0.248 (−0.485 to −0.010)	0.04	−0.206 (−0.466 to 0.054)	0.12
Trash burning	0.123 (−0.184 to 0.429)	0.43	0.189 (−0.133 to 0.511)	0.25
Traffic flow				
No traffic or stopped vehicle	0.0	NA	0.0	NA
Idling vehicle	−0.084 (−0.383 to 0.216)	0.58	−0.126 (−0.442 to 0.190)	0.44
Light (< 2 cars/min)	0.110 (0.014 to 0.206)	0.03	0.112 (0.011 to 0.213)	0.03
Medium (< 10 cars/min)	0.247 (0.138 to 0.357)	< 0.001	0.247 (0.131 to 0.362)	< 0.001
Heavy moving	0.370 (0.242 to 0.498)	< 0.001	0.383 (0.248 to 0.518)	< 0.001
Congested/stopped heavy traffic	0.537 (0.350 to 0.724)	< 0.001	0.528 (0.331 to 0.725)	< 0.001
Biomass stoves				
No stove	0.0	NA	0.0	NA
Single charcoal stove	0.116 (0.012 to 0.220)	0.03	0.104 (−0.006 to 0.214)	0.06
Multiple charcoal stoves	0.225 (0.088 to 0.363)	0.001	0.243 (0.099 to 0.387)	0.001
Single wood stove	0.277 (0.051 to 0.504)	0.02	0.287 (0.049 to 0.524)	0.02
Multiple wood stoves	0.677 (0.505 to 0.849)	< 0.001	0.818 (0.638 to 0.999)	< 0.001
Road surface				
Paved	0.0	NA	0.0	NA
Paved broken	0.213 (0.049 to 0.377)	0.011	0.243 (0.072 to 0.414)	0.005
Packed dirt	0.062 (−0.031 to 0.156)	0.19	0.036 (−0.063 to 0.135)	0.47
Loose dirt	0.223 (0.078 to 0.367)	0.003	0.264 (0.111 to 0.416)	0.001
Meteorologic factors				
Wind speed (miles/hr)	−0.011 (−0.024 to 0.003)	0.12	−0.036 (−0.050 to −0.023)	< 0.001
RH	1.590 (1.074 to 2.105)	< 0.001	3.117 (2.560 to 3.674)	< 0.001
Ln(hours since rain + 1)	0.132 (0.075 to 0.190)	< 0.001	0.150 (0.043 to 0.256)	0.006

NA, not applicable. Model 1 is adjusted for neighborhood average (estimated as average of smoothed concentrations at all fixed sites) and Model 2 is without this variable. See “Materials and Methods” for details.
